# Comprehensive ESI-Q TRAP-MS/MS based characterization of metabolome of two mango (*Mangifera indica* L) cultivars from China

**DOI:** 10.1038/s41598-020-75636-y

**Published:** 2020-11-18

**Authors:** Lin Tan, Zhiqiang Jin, Yu Ge, Habibullah Nadeem, Zhihao Cheng, Farrukh Azeem, Rulin Zhan

**Affiliations:** 1 Haikou Experimental Station, Chinese Academy of Tropical Agricultural Sciences, Haikou, 571101 China; 2 Institute of Tropical Bioscience and Biotechnology, Chinese Academy of Tropical Agricultural Sciences, Haikou, Hainan China; 3 Department of Bioinformatics and Biotechnology, Government College University Faisalabad, Faisalabad, Pakistan

**Keywords:** Plant sciences, Plant molecular biology

## Abstract

Polyphenols based bioactive compounds from vegetables and fruits are known for impressive antioxidant activity. Ingestion of these antioxidants may promote human health against cardiovascular diseases and cancer. Mango is a popular tropical fruit with special taste, high nutritional value and health-enhancing metabolites. The aim was to investigate the diversity of phytochemicals between two mango cultivars of china at three stages of fruit maturity. We used ESI-QTRAP-MS/MS approach to characterize comprehensively the metabolome of two mango cultivars named Hongguifei (HGF) and Tainong (TN). HPLC was used to quantify selected catechin based phenolic compounds. Moreover, real-time qPCR was used to study the expression profiles of two key genes (*ANR* and *LAR*) involved in proanthocyanidin biosynthesis from catechins and derivatives. A total of 651 metabolites were identified, which include at least 257 phenolic compounds. Higher number of metabolites were differentially modulated in peel as compared to pulp. Overall, the relative quantities of amino acids, carbohydrates, organic acids, and other metabolites were increased in the pulp of TN cultivar. While the contents of phenolic compounds were relatively higher in HGF cultivar. Moreover, HPLC based quantification of catechin and derivatives exhibited cultivar specific variations. The *ANR* and *LAR* genes exhibited an opposite expression profile in both cultivars. Current study is the first report of numerous metabolites including catechin-based derivatives in mango fruit. These findings open novel possibilities for the use of mango as a source of bioactive compounds.

## Introduction

Fruits of tropical and subtropical regions are appreciated as energy suppliers, as well as for the presence of health-enhancing metabolites. Plant origin secondary metabolites are the focus of research for numerous health-beneficial properties and antioxidant activities. Mango (*Mangifera indica* (L.) Lam.) is an important tropical fruit and rank fifth in global production (55.6 million tonnes/year) after banana, apple, grapes, and oranges^1^. Numerous cultivars of mango are found worldwide, which show variations in fruit peel color, size, shape, and composition^2,3^. Apart from being consumed fresh, mangoes are also used to make desserts, juices, pickles, marmalades and jam^4^. During processing, a significant portion of the fruit is removed, which generates millions of tons of mango waste every year. However, the peel of mango fruit may be interesting for the presence of high levels of health-promoting compounds^2,4^. It seems in line with the efforts to explore the cost-effective potential of agri-waste for industrial use or to reduce its negative effects on the environment. The identification and reclamation of important metabolites from mango or its byproducts is a difficult task and its completion would promote the revaluation of mango as a natural source of antioxidants/bioactive compounds. In this regard, large scale metabolite profiling seems a promising way to explore metabolite potential of mango fruit.

Proanthocyanidins (PAs) are oligo/polymeric flavonoids that are naturally present in many vegetables, nuts, seeds and fruits^5^. Flavan-3-ols constitute the structural units of PAs and consist of C6-C3-C6 based flavonoid skeleton. There are two most common forms of these metabolites as 2,3-*cis*-(–)-epicatechin and 2,3-*trans*-(+)-catechin^6^. Flavan-3-ols (as monomers or as PAs) promote plant resistance against various biotic and abiotic stresses^7^. These metabolites possess numerous pharmacological properties, hence involved in scavenging free radicals, antimicrobial, antioxidant, anti-nutritional, anti-cancer and cardiac protection activities^8,9^. Quantitative and qualitative differences in the phytochemical profiles of mango cultivars may contribute to distinguishing their health-promoting properties.

The biochemical composition of fruits (sugars, organic acids, flavonoids, etc.) predominantly influences the consumer preference for visual features and taste. Several researchers have investigated the nutritional composition of mangoes^10–12^. However, very limited information is available about the identities of metabolites, which govern important properties of this fruit. In addition, there is a lack of studies for comprehensive identification, documentation, and quantification of flavonoids and other secondary metabolites. Instead of thoroughly evaluating all of the phytochemicals, researchers have attempted to study only particular metabolites of mango^10,13,14^. Recent advancements in widely-targeted metabolomics (supported by techniques like LC–MS/MS) have made possible a prompt and ultra-sensitive detection of a huge number of metabolites^15,16^. The liquid chromatography-tandem mass spectrometry is employed in the current study to identify and detect relative quantities of metabolites from two mango cultivars exhibiting contrasting features in terms of shape, size, taste and peel color^2^. This study aims at the revelation of the metabolic variations between two mango cultivars (from China) with distinct features^2,17^ and offers valuable data for appraising its nutritional importance in industrial utilization and breeding strategies.

## Results and discussion

### Metabolic profiling

Previous studies have reported the quantification of individual metabolite classes in mango pulp and peel^10,18–24^. In most of these studies, a standard metabolite was used to identify an exact compound or a relevant group of metabolites. However, based on these studies it was difficult to envisage comprehensive metabolic dynamics in mango. In current study, a total of 651 metabolites were annotated/ identified in three growth stages (Table S1), which include 54 nucleotides and their derivatives; 21 carbohydrates and their derivatives; 99 amino acids and their derivatives; 67 lipids and their derivatives; 6 indole derivatives; 8 alcohols and polyols; 2 terpenoids; 5 alkaloids; 21 vitamins and their derivatives; 72 organic acids; 257 phenolic compounds and 38 other metabolites.

### Principal component analysis (PCA) for metabolite profiles

**Figure 1:**
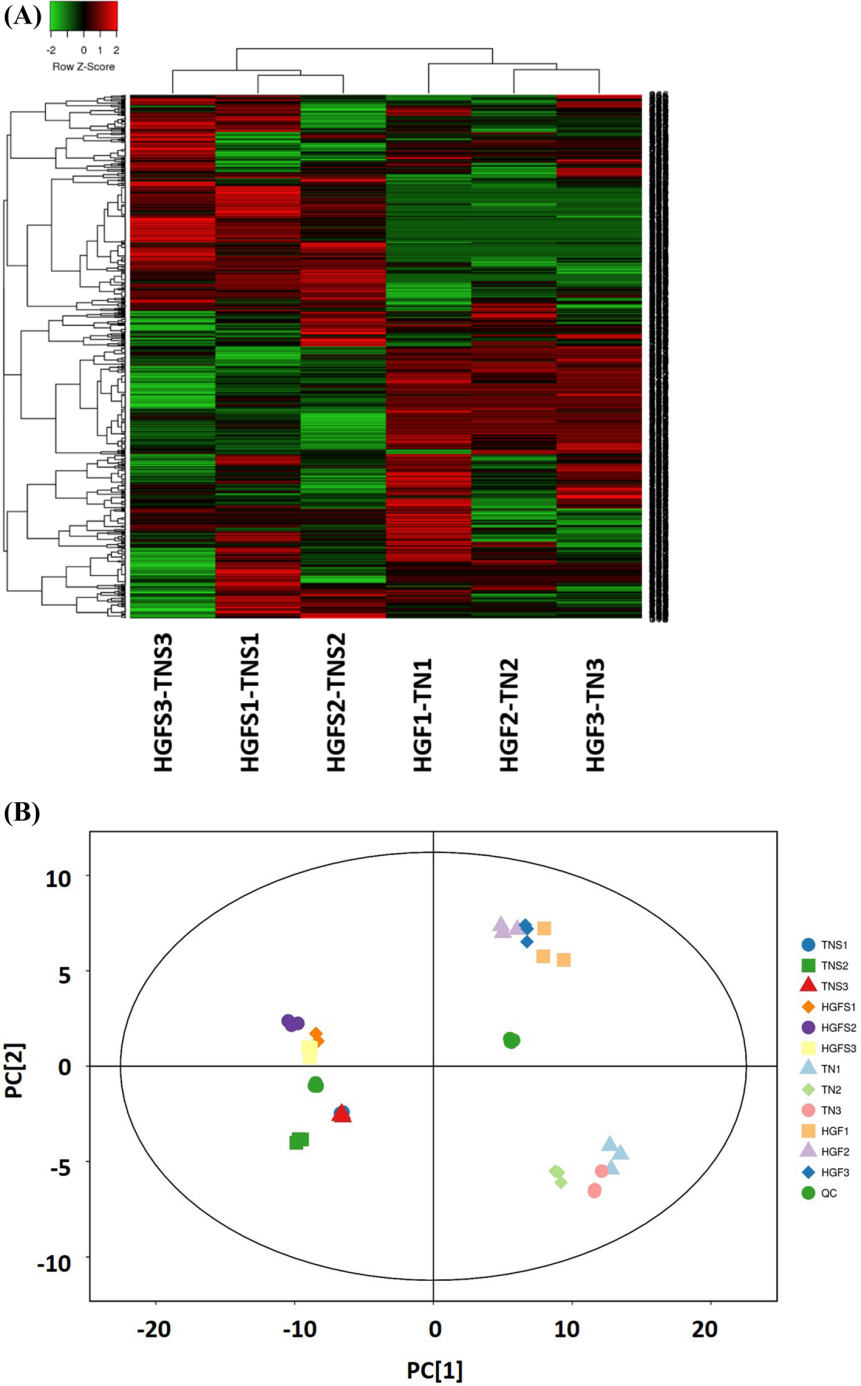
HCA and PCA analysis of relative metabolite variations in peel and pulp samples of both cultivars. Both the HCA and PCA analysis was performed using all the metabolites annotated in current study. (**A**) Heat map for HCA. An online tool (heatmapper)^25^ was used to visualize the metabolite variations. The complete linkage hierarchical clustering was used for normalization. Each column represents a pairwise comparison of metabolites from both cultivars at a particular stage, while each row represents a metabolite. The red color is an indication of a higher concentration in HGF and green color represents a higher concentration of the metabolite in TN. (**B**) PC1 and PC2 score plots for pulp and peel between both cultivars. TN1, TN2, TN3, HGF1, HGF2, and HGF3 represent pulp samples of TN and HGF cultivars at first, second and third stage respectively. Similarly, TNS1, TNS2, TNS3, HGFS1, HGFS2, and HGFS3 represent peel samples of TN and HGF cultivars at first, second and third stage respectively. QC, mix represents quality control samples.

To assess relative variations in metabolic profiles (for 651 metabolites), we used multivariate statistics. Hierarchical cluster analysis (HCA), for relative differences in accumulation patterns at three growth stages, arranged metabolites from both cultivars into three groups (Fig. 1A). Peel and pulp samples exhibit distinct metabolite profiles in terms of upregulated or downregulated compounds in a cultivar and growth stage-specific manner (Fig. 1A, Table S2). The metabolites in peel at stages 1 and 2 are clustered in the same column as compared to third stage metabolites. On the other hand, metabolites at stages 2 and 3 are clustered in a similar column for pulp samples as compared to the first stage. It suggests the diversity of metabolites in peel samples at the third stage of fruit growth.

PCA is extensively applied in chemometric experiments to extract and rationalize important facts from biological systems with multivariate descriptions. By using this analysis, we can determine the core arrangement of variables in terms of principal components. According to PCA plots (Fig. 1B), the QC samples (mix) formed a close cluster, which indicates the similarity of metabolic profiles and stability/repeatability of analysis. Consistently, based on the PC1, a clear separation could be observed between the peel and pulp metabolites. Additionally, both cultivars are clearly distinguished by PC2 (Fig. 1B). This finding suggests the existence of distinct metabolic programs in peels and pulps. Moreover, both cultivars exhibit discrete metabolites in respective tissues that could be the basis of their contrasting features.

### Partial least-squares discriminant analysis (PLS-DA) for differential metabolites

**Figure 2:**
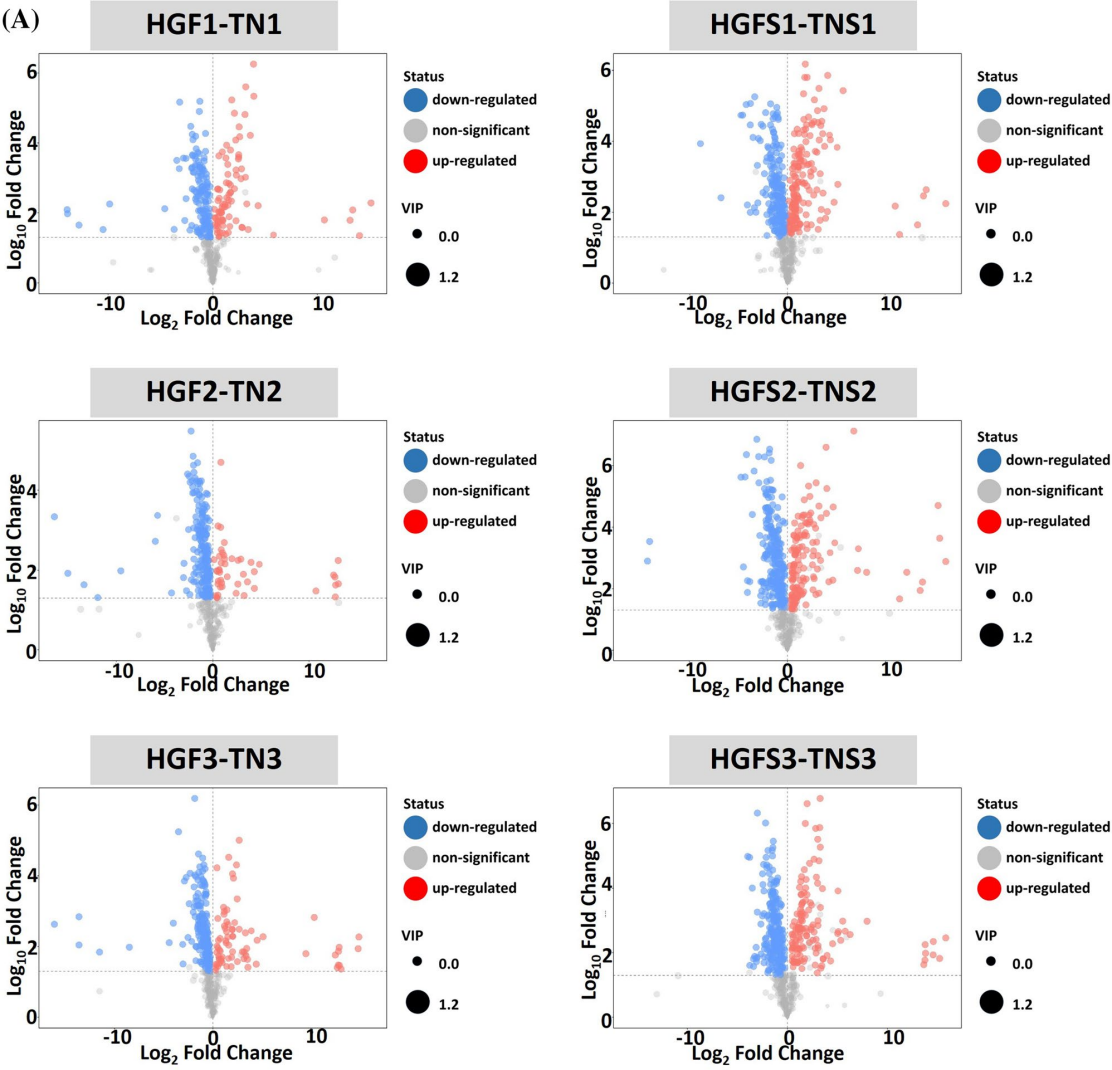

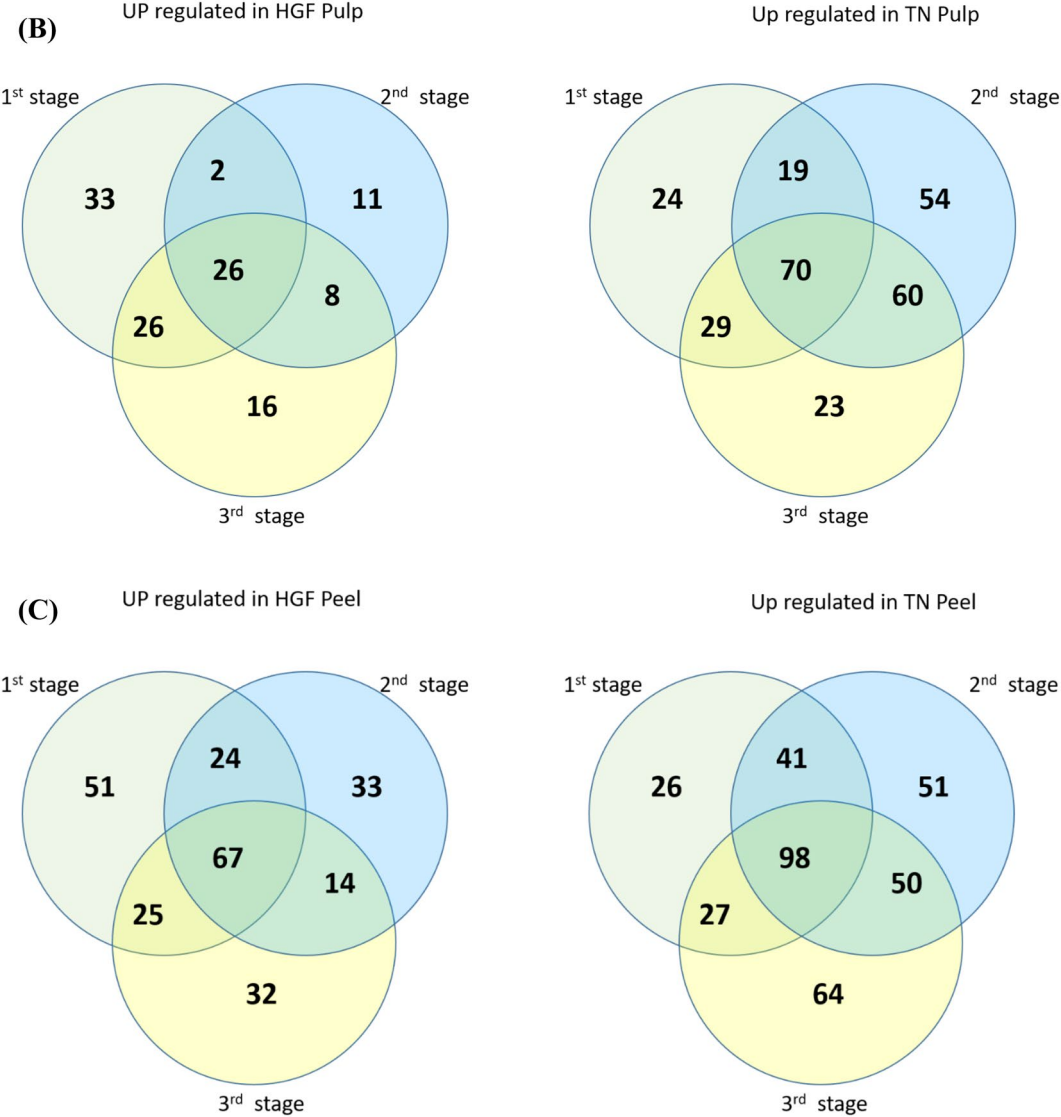
Volcano plots and Ven diagrams for differentially expressed metabolites in peel and pulp samples. (**A**) volcano plots; (**B**) Ven diagram of pulp samples; (**C**) Ven diagram of peel samples. TN1, TN2, TN3, HGF1, HGF2, and HGF3 represent pulp samples of TN and HGF cultivars at first, second and third stage respectively. Similarly, TNS1, TNS2, TNS3, HGFS1, HGFS2, and HGFS3 represent peel samples of TN and HGF cultivars at first, second and third stage respectively.

For the estimation of potential contributions of metabolites in cultivar-specific features, pairwise comparisons were performed using OPLS-DA models among three stages of mango fruit for pulp and peel (as inter-cultivar pairs). As a result, higher predictability (Q^2^) and strong goodness of fit (R^2^X, R^2^Y) were observed for these models. (Table S3; Fig. S1). All the values of R^2^X, R^2^Y and Q^2^ in OPLS-DA are above 0.7 and even many of them are close to 1 (Table S3), indicating the models is very good. Besides, R^2^Y in the permutation test of OPLS-DA of each group is very close to 1 (Fig. S1), suggesting the established model conforms to the real situation of sample data. The Q^2^ in each model is very close to 1, which shows that the model can well explain the difference between the two groups of samples. The model has no over fitting phenomenon and is very stable. For further improving our understanding of metabolic variations, we performed a differential metabolite screening among all detected metabolites for fold-change and the projection scores or VIP values. The metabolites were considered differentially expressed if the *p* value was less than 0.05 and the VIP value was greater than 1. The results of this screening are presented using Volcano plots in Fig. 2A and summarized in Table S2.

To study the cultivar-specific relative quantification of metabolites in the pulp, a comparison was drawn between pulp metabolites of HGF and TN cultivars. At 1st stage of fruit growth, there were 231 differential metabolites (87 and 144 up-regulated in HGF and TN, respectively), 252 metabolites (47 and 205 up-regulated in HGF and TN, respectively) at 2nd stage, and 261 metabolites (76 and 185 up-regulated in HGF and TN, respectively) at 3rd stage (Fig. 2B, HGF1-TN1, HGF2-TN2, HGF3-TN3). In other words, a higher number of metabolites were up-regulated in TN cultivar as compared to HGF.

#### Metabolites in pulps

To address the prospective importance of metabolite concentration in fruit maturity features, it was observed that sebacate and *ρ*-hydroxyphenyl acetic acid (organic acids); indole and epigallocatechin (catechin derivative) were present in very high amounts (> 10 log_fold) in TN pulp (Table S2, represented by blue color in HGF3-TN3). On the other hand in HGF cultivar, 6-hydroxynicotinic acid (vitamins); rosinidin *o*-hexoside (Anthocyanins); protocatechuic aldehyde (catechin derivatives); syringic acid, 1-*o*-beta-d-glucopyranosyl sinapate (hydroxycinnamoyl derivatives); chrysin *o*-hexoside, chrysin 5-*o*-glucoside (toringin) (flavone); *o*-feruloyl quinic acid (quinate and its derivatives); eriodictyol *c*-hexoside (flavone *c*-glycosides); and aromadedrin (flavonol) were present in very high amounts (> 10 log_fold). Overall, the relative quantities of amino acids, carbohydrates, organic acids, and other metabolites were higher in the pulp of TN cultivars. While the contents of phenolic compounds were relatively higher in HGF cultivar. It explains better sweetness/taste of TN and better quality of HGF pulp^17^. Among others, syringetin (a flavonol) was detected only at the 2nd stage as ~ 10.71 log_fold higher contents in HGF as compared to TN. This compound has never been reported in mango. Besides, it was just reported in grape and wine^26^. This metabolite can induce human osteoblast differentiation through bone morphogenetic protein‐2/extracellular signal‐regulated kinase 1/2 pathway^26^. It can also enhance radio-sensitivity more effectively in cancer cells than in normal cells through enhancement of the caspase-3-mediated apoptosis pathway^27^. Moreover, growth stage or cultivar-specific variations in metabolite contents may provide important information in identifying respective molecular markers for mango fruit selection at different growth stages (Fig. 2B).

#### Metabolites in peels

In a comparison of peel samples between HGF and TN cultivars, the upregulated metabolites were as follows: 357 at 1st stage (165 HGF vs 192 TN), 379 at 2nd (138 HGF vs 241 TN) and 375 at 3rd (136 HGF vs 239 TN) (Fig. 2C). However, in the comparison of differential metabolites between growth stages, a higher number of metabolites were upregulated in lateral growth stages. Likewise pulp metabolites, TN peel exhibited up-regulation of a higher number of metabolites as compared to peel of HGF (Fig. 2C).

With a view to exploit the potential of mango peel as a source of valuable metabolites that vary between these two cultivars at very high levels (> 13 log_fold), following metabolites were detected in HGF peel (as compared to TN): DIMBOA glucoside (cyclic hydroxamic acid); spinacetin (flavone); sissotrin (isoflavone); *C*-hexosyl-chrysoeriol *o*-hexoside (flavone *C*-glycosides); cucurbitacin D (terpenoids); gentisic acid (benzoic acid derivatives); Vanillic acid (hydroxycinnamoyl derivatives); *N*-sinapoyl hydroxycoumarin (coumarins) (Table S2, HGFS3-TNS3). These metabolites mainly constituted flavonoids and may be considered as representative differential metabolites for the different peel colors in both cultivars (Table S2). DIMBOA glucoside represented the most upregulated compound in the peel of HGF cultivar (> 15 log_fold). It belongs to benzoxazinoids (a group of cyclic hydroxamic acids), which are found prevalently in the members of family Poaceae. This metabolite has been reported from *Secale cereale* L., *Triticum aestivum* L. and *Zea mays* L.^28^. The benzoxazinoid derivatives were discovered in nature in the 1950s and have been attracting significant scientific interest in nutrition and pharmaceutics during the past decade^29^. Benzoxazinoid hydroxamic acids have been reported that exhibit phytotoxic activities, playing a significant role in plant defense against fungi, bacteria, insects, and participating in allelopathy mechanisms^28,30^. In this context, it is the first report of DIMBOA-glucoside outside the grass family. Similarly, cucurbitacins are identified as tetracyclic triterpenoids and belong to the Cucurbitaceae family. They are known to have diverse pharmacological activities including antimicrobial activities, anti-inflammatory, antitumor and cardiovascular properties^31^. Cucurbitacin D (Table S2, HGFS3-TNS3; > 13 log_fold) effectively inhibits glucose uptake and lactate production in metastatic prostate cancer cells via modulating glucose metabolism^32^. These findings open novel possibilities for the use of mango peel as a source of plant bioactive compounds.

### Differential metabolic pathways

To obtain detailed pathway information, the Kyoto Encyclopedia of Genes and Genomes (KEGG) database (https://www.genome.jp/kegg/) was used to map the differential metabolites between both cultivars (Fig. S2, Table S4). The identified metabolic pathways with differential metabolites compared between pulps or peels of both cultivars are shown in Fig. S2A and S2B. These pathways were mainly involved in aminoacyl-tRNA biosynthesis, purine metabolism, glucosinolate biosynthesis, phenylpropanoid biosynthesis (PP), flavonoid biosynthesis and pathways involved in amino acid metabolism (Table S4). These pathways are also involved in the metabolism of plant secondary metabolites. Flavonoids are considered one of the major contributors to crucial features of fruits. The PP pathway in pulp and peel samples differed between both cultivars for differentially expressed metabolites (DEMs) (Fig. S2A and S2B). It is possible that the expression of flavonoid biosynthesis-related genes could be related to genotypic differences.

### Phenolic compounds

As the phenolic compounds constituted the largest group of metabolites identified in this analysis, so we decided to analyze this group in detail (Table 1). Previously, a variable but limited number of phenolic compounds were reported in mango fruit^10,19,33^. Mango fruit generally contains two groups of phenolic acids i.e., hydroxybenzoic and hydroxycinnamic acid derivatives. In literature, hydroxybenzoic acids like protocatechuic acid, vanillic acid, *ρ*-hydroxybenzoic acid, gallic acid, syringic acid and hydroxycinnamic acids like caffeic acid, chlorogenic acid, ferulic acid, and *ρ*-coumaric acid have been reported in mango. Although, the metabolic content and type vary with geographical location, plant age and variety^2^. In current study, the majority of metabolites are reported for the first time in mango (Table 1).

**Table 1:** All the phenolic compounds identified in mango fruit (pulp and peel) of both cultivars during three growth stages.

Sr. no	Class	Metabolite	Precursor ions Q1 (Da)	Product ions Q3 (Da)	Rt. (min)	Molecular weight (Da)	Ionization model	KEGG.ID
1	Anthocyanins	Cyanidin 3-*O*-glucosyl-malonylglucoside	697.1	696.9	2.24	697.1	[M+H]^+^	–
2	Anthocyanins	Delphinidin 3-*O*-glucoside (Mirtillin)	465.1	303.1	2.26	465.1	[M+H]^+^	C12138
3	Anthocyanins	Pelargonin	595	271.8	2.38	595	[M+H]^+^	C08725
4	Anthocyanins	Petunidin 3-*O*-glucoside	479	317	2.56	479	[M+H]^+^	C12139
5	Anthocyanins	Cyanidin 3-*O*-glucoside (Kuromanin)	449.1	287.3	2.59	449.1	[M+H]^+^	C08604
6	Anthocyanins	Cyanidin *O*-syringic acid	465.1	285.3	2.59	466.1	[M−H]^−^	–
7	Anthocyanins	Pelargonidin 3-*O*-*β*-D-glucoside (Callistephin chloride)	433.1	271	2.83	433.1	[M+H]^+^	–
8	Anthocyanins	Malvidin 3-*O*-glucoside (Oenin)	493.2	331.6	2.92	493.2	[M+H]^+^	C12140
9	Anthocyanins	Delphinidin	303	149.3	2.98	303.24	[M+H]^+^	C05908
10	Anthocyanins	Peonidin *O*-malonylhexoside	547.1	503.4	3	548.1	[M−H]^−^	–
11	Anthocyanins	Cyanidin *O*-diacetyl-hexoside-*O*-glyceric acid	619.1	531.3	3.26	620.1	[M−H]^−^	–
12	Anthocyanins	Rosinidin *O*-hexoside	477.1	315.6	3.32	477.1	[M+H]^+^	–
13	Anthocyanins	Cyanidin	287	231.6	3.54	287.24	[M+H]^+^	C05905
14	Anthocyanins	Pelargonidin	271	215.1	3.85	271.24	[M+H]^+^	C05904
15	Anthocyanins	Peonidin	301.1	273.6	3.95	301.1	[M+H]^+^	C08726
16	Benzoic acid derivatives	Anthranilate *O*-hexosyl-*O*-hexoside	460.1	118.2	0.78	461.1	[M−H]^−^	–
17	Benzoic acid derivatives	Gallic acid	169	122.8	1.74	170.022	[M−H]^−^	C01424
18	Benzoic acid derivatives	2,5-dihydroxy benzoic acid *O*-hexside	315.1	152.1	1.84	316.1	[M−H]^−^	–
19	Benzoic acid derivatives	Gallic acid *O*-Hexoside	331	313.7	2.02	332	[M−H]^−^	–
20	Benzoic acid derivatives	Syringic acid *O*-glucoside	359.1	197.1	2.26	360.1	[M−H]^−^	–
21	Benzoic acid derivatives	2,5-dihydroxybenzoic acid (Gentisic acid)	153	108.1	2.5	154.027	[M−H]^−^	C00628
22	Benzoic acid derivatives	2,4-Dihydroxybenzoic acid	153	109	2.75	154.027	[M−H]^−^	–
23	Benzoic acid derivatives	Methyl gallate	183	124.1	3.14	184.0372	[M−H]^−^	–
24	Benzoic acid derivatives	*p*-Aminobenzoate	137.3	94.3	3.14	136.3	[M+H]^+^	C00568
25	Benzoic acid derivatives	4-Hydroxybenzaldehyde	121	91.9	3.72	122.037	[M−H]^−^	C00633
26	Benzoic acid derivatives	Ethyl gallate	197.1	124.1	3.87	198.0528	[M−H]^−^	–
27	Benzoic acid derivatives	Vanillin	151	136.1	4	152.0473	[M−H]^−^	C00755
28	Benzoic acid derivatives	8-Methyl-2-oxo-4-phenyl-2*H*-chromen-7-yl 4-(hexyloxy)benzoate	457.2	191.5	4.48	456.2	[M+H]^+^	–
29	Benzoic acid derivatives	Benzoic acid	121	77	4.57	122.0368	[M−H]^−^	C00180
30	Catechin derivatives	( +)-Gallocatechin (GC)	307	248.1	2.27	306.074	[M+H]^+^	C12127
31	Catechin derivatives	Protocatechuic acid *O*-glucoside	315.1	153.2	2.44	316.1	[M−H]^−^	–
32	Catechin derivatives	Protocatechuic acid	153.1	109.1	2.48	154.027	[M−H]^−^	C00230
33	Catechin derivatives	Epigallocatechin (EGC)	307	139.1	2.73	306	[M+H]^+^	C12136
34	Catechin derivatives	Epigallocatechin (EGC)	305	125	2.76	306	[M−H]^−^	C12136
35	Catechin derivatives	Catechin	291.1	139.1	2.99	290.079	[M+H]^+^	C06562
36	Catechin derivatives	Protocatechuic aldehyde	137.1	137	3.06	138.032	[M−H]^−^	C16700
37	Catechin derivatives	*L*-Epicatechin	289	78.8	3.18	290.3	[M−H]^−^	C09727
38	Catechin derivatives	Epigallate catechin gallate (EGCG)	459	139.1	3.32	458.085	[M+H]^+^	C09731
39	Catechin derivatives	Catechin–catechin–catechin	865.1	407.2	3.44	866.1	[M−H]^−^	–
40	Catechin derivatives	Epicatechin-epiafzelechin	561.1	271.3	3.61	562.1	[M−H]^−^	–
41	Catechin derivatives	Epicatechin gallate (ECG)	441.3	289.1	3.89	442.3	[M−H]^−^	–
42	Cholines	Choline	104.1	60.2	0.76	103.1	[M+H]^+^	C00114
43	Cholines	*O*-Phosphocholine	184	83.2	0.78	183	[M+H]^+^	C00588
44	Cholines	sn-Glycero-3-phosphocholine	258.2	125.2	0.78	258.2	[M+H]^+^	C00670
45	Cholines	Acetylcholine	147.1	88	0.85	146.1181	[M+H]^+^	C08201
46	Coumarins	Esculetin (6,7-dihydroxycoumarin)	177	133.1	3.24	178.027	[M−H]^−^	C09263
47	Coumarins	Daphnetin	179	179	3.31	178.027	[M+H]^+^	C03093
48	Coumarins	*O*-Feruloyl 2-hydroxylcoumarin	339.1	177.5	3.32	338.1	[M+H]^+^	–
49	Coumarins	*O*-Feruloyl 3-hydroxylcoumarin	339.1	177.5	3.34	338.1	[M+H]^+^	–
50	Coumarins	*N*-sinapoyl hydroxycoumarin	369.1	207.5	3.82	368.1	[M+H]^+^	–
51	Coumarins	*O-*Feruloyl 4-hydroxylcoumarin	339.1	177.5	3.88	338.1	[M+H]^+^	–
52	Coumarins	Scopoletin (7-Hydroxy-5-methoxycoumarin)	193.1	178.1	4	192.042	[M+H]^+^	C01752
53	Coumarins	Scoparone	207.1	207.1	4.73	206.058	[M+H]^+^	C09311
54	Coumarins	3,4-Dihydrocoumarin	149.2	107	5.63	148.052	[M+H]^+^	C02274
55	Coumarins	6-MethylCoumarin	161	105.1	5.96	160.052	[M+H]^+^	–
56	Flavanone	Afzelechin (3,5,7,4′-Tetrahydroxyflavan)	275	139.1	3.4	274.084	[M+H]^+^	C09320
57	Flavanone	Hesperetin 5-*O*-glucoside	463.1	301.1	3.85	464.132	[M−H]^−^	–
58	Flavanone	Hesperetin *O*-malonylhexoside	549.2	387.3	3.99	550.2	[M−H]^−^	–
59	Flavanone	Naringenin 7-*O*-glucoside (Prunin)	433.1	122.9	4.22	434.1213	[M−H]^−^	C09099
60	Flavanone	Naringenin *O*-malonylhexoside	521	317.6	4.5	520	[M+H]^+^	–
61	Flavanone	Liquiritigenin	255	119	5.16	256.074	[M−H]^−^	C09762
62	Flavanone	Butein	271.1	135.1	5.49	272.069	[M−H]^−^	C08578
63	Flavanone	Phloretin	273.1	167.1	5.56	274.084	[M−H]^−^	C00774
64	Flavanone	Naringenin chalcone	273.1	153.1	5.57	272.069	[M+H]^+^	C06561
65	Flavanone	Naringenin	273.1	153.1	5.59	272.0685	[M+H]^+^	C00509
66	Flavanone	Isoliquiritigenin	255	119.1	6.09	256.074	[M−H]^−^	C08650
67	Flavanone	7-*O*-Methyleriodictyol	301.1	135.1	6.28	302.079	[M−H]^−^	–
68	Flavanone	4′-Hydroxy-5,7-dimethoxyflavanone	299.1	74.8	6.78	300.1	[M−H]^−^	–
69	Flavanone	Isosakuranetin (4′-Methylnaringenin)	287.1	161.1	6.81	286.084	[M+H]^+^	C05334
70	Flavanone	Pinocembrin (Dihydrochrysin)	257.1	153	7.05	256.074	[M+H]^+^	C09827
71	Flavanone	Xanthohumol	355.2	178.9	8.4	354.147	[M+H]^+^	C16417
72	Flavone	Chrysoeriol *O*-hexosyl-*O*-malonylhexoside	709.1	547.3	2.49	710.1	[M−H]^−^	–
73	Flavone	Acacetin *O*-acetyl hexoside	487.1	283.2	2.63	488.1	[M−H]^−^	–
74	Flavone	Luteolin *O*-hexosyl-*O*-hexosyl-*O*-hexoside	771.1	609.5	2.83	772.1	[M−H]^−^	–
75	Flavone	Selgin 5-*O*-hexoside	479.1	302.8	3.52	478.1	[M+H]^+^	–
76	Flavone	Tricin *O*-sinapic acid	535	329.4	3.75	536	[M−H]^−^	–
77	Flavone	Tricin *O*-saccharic acid	521.1	329.3	3.81	522.1	[M−H]^−^	–
78	Flavone	Luteolin 7-*O*-glucoside (Cynaroside)	449.1	287.1	3.87	448.101	[M+H]^+^	C03951
79	Flavone	Chrysoeriol *O*-acetylhexoside	503.1	341.3	3.94	504.1	[M−H]^−^	–
80	Flavone	Apigenin 7-*O*-neohesperidoside (Rhoifolin)	579.2	271.1	4.01	578.1636	[M+H]^+^	C12627
81	Flavone	Apigenin 7-rutinoside (Isorhoifolin)	579.2	271.1	4.01	578.1636	[M+H]^+^	–
82	Flavone	Chrysoeriol *O*-rhamnosyl-*O*-glucuronic acid	621.1	299.4	4.07	622.1	[M−H]^−^	–
83	Flavone	Tricin di-*O*-hexoside	655.2	331.7	4.16	654.2	[M+H]^+^	–
84	Flavone	Syringetin 5-*O*-hexoside	509.2	347.6	4.17	508.2	[M+H]^+^	–
85	Flavone	Syringetin 7-*O*-hexoside	509.3	283.6	4.17	508.3	[M+H]^+^	–
86	Flavone	Selgin *O*-hexosyl-*O*-hexoside	641.1	479.5	4.39	640.1	[M+H]^+^	–
87	Flavone	Spinacetin	347	288	4.41	346	[M+H]^+^	–
88	Flavone	Chrysoeriol *O*-malonylhexoside	549.1	301.7	4.55	548.1	[M+H]^+^	–
89	Flavone	Tricin *O*-malonylhexoside	579.1	331.7	4.56	578.1	[M+H]^+^	–
90	Flavone	7,4′-Dihydroxyflavone	255.1	137.1	4.57	254.058	[M+H]^+^	C12123
91	Flavone	Apigenin 4-*O*-rhamnoside	417.1	270.9	4.9	416.111	[M+H]^+^	–
92	Flavone	Chrysoeriol *O*-sinapoylhexoside	669	301.6	4.92	668	[M+H]^+^	–
93	Flavone	Chrysin 5-*O*-glucoside (Toringin)	417.1	255.7	5	416.1	[M+H]^+^	–
94	Flavone	Luteolin	287.1	287.1	5	286.1	[M+H]^+^	C01514
95	Flavone	Acetyl-eriodictyol *O*-hexoside	491.1	287.3	5.16	492.1	[M−H]^−^	–
96	Flavone	Chrysin *O*-malonylhexoside	503	255.6	5.24	502	[M+H]^+^	–
97	Flavone	Butin	273.1	153.1	5.59	272.069	[M+H]^+^	C09614
98	Flavone	Apigenin	271.1	215.1	5.63	270.0528	[M+H]^+^	C01477
99	Flavone	Tricin	331.1	315.8	5.74	330.1	[M+H]^+^	–
100	Flavone	Chrysoeriol	301.1	286.1	5.77	300.0634	[M+H]^+^	C04293
101	Flavone	Tricin 7-*O*-acetylglucoside	535.3	487.2	5.78	534.3	[M+H]^+^	–
102	Flavone	Amentoflavone	539.1	403	5.88	538.09	[M+H]^+^	C10018
103	Flavone	Baicalein (5,6,7-Trihydroxyflavone)	269.1	251.1	5.94	270.053	[M−H]^−^	C10023
104	Flavone	Chrysin	255.1	69.7	6.95	254.0579	[M+H]^+^	C10028
105	Flavone	sakuranetin	287.1	287.1	6.96	286.084	[M+H]^+^	C09833
106	Flavone	Acacetin	283.1	268	7.06	284.069	[M−H]^−^	C01470
107	Flavone	Nobiletin	403.1	373.1	7.06	402.132	[M+H]^+^	C10112
108	Flavone	Velutin	313.1	298.3	7.22	314.1	[M−H]^−^	–
109	Flavone	Tangeretin	373.1	373.1	7.54	372.121	[M+H]^+^	C10190
110	Flavone C-glycosides	8-*C*-hexosyl-hesperetin *O*-hexoside	627.1	430	2.78	626.1	[M+H]^+^	–
111	Flavone C-glycosides	6-*C-*hexosyl-luteolin *O*-hexoside	611.1	329	3.09	610.1	[M+H]^+^	–
112	Flavone C-glycosides	Eriodictiol *C*-hexosyl-*O*-hexoside	613.1	300.3	3.09	612.1	[M+H]^+^	–
113	Flavone C-glycosides	*C*-hexosyl-chrysoeriol *O*-hexoside	625.2	463.6	3.37	624.2	[M+H]^+^	–
114	Flavone C-glycosides	Eriodictyol *C*-hexoside	449.1	329.3	3.37	450.1	[M−H]^−^	–
115	Flavone C-glycosides	6-*C*-hexosyl-hesperetin *O*-hexoside	627.1	447.3	3.41	626.1	[M+H]^+^	–
116	Flavone C-glycosides	Luteolin 6-*C*-glucoside	449.1	300	3.45	448.1	[M+H]^+^	–
117	Flavone C-glycosides	Luteolin *C*-hexoside	449.1	329.6	3.45	448.1	[M+H]^+^	–
118	Flavone C-glycosides	*C*-hexosyl-luteolin *O*-hexosyl-*O*-salicylic acid	731.1	431.5	3.55	730.1	[M+H]^+^	–
119	Flavone C-glycosides	*C*-hexosyl-apigenin *O*-pentoside	565.1	397.8	3.6	564.1	[M+H]^+^	–
120	Flavone C-glycosides	di-*C*,*C-*hexosyl-apigenin	595.1	415.4	3.68	594.1	[M+H]^+^	–
121	Flavone C-glycosides	Vitexin 2*″-O*-*β*-L-rhamnoside	579	433.1	3.68	578.164	[M+H]^+^	C12628
122	Flavone C-glycosides	*C*-hexosyl-luteolin *O*-*p*-coumaroylhexoside	757.2	757	3.73	756.2	[M+H]^+^	–
123	Flavone C-glycosides	8-*C*-hexosyl-luteolin *O*-hexoside	611.1	299.8	3.75	610.1	[M+H]^+^	–
124	Flavone C-glycosides	Apigenin *C*-glucoside	433.1	271.7	3.78	432.1	[M+H]^+^	–
125	Flavone C-glycosides	Isovitexin	431.1	431.1	3.79	432.1056	[M−H]^−^	C01714
126	Flavone C-glycosides	Naringenin *C-*hexoside	435.1	339.7	3.79	434.1	[M+H]^+^	–
127	Flavone C-glycosides	Acacetin *C*-hexoside	447.1	298	3.84	446.1	[M+H]^+^	–
128	Flavone C-glycosides	*O*-methylnaringenin C-pentoside	419.1	383.9	3.84	418.1	[M+H]^+^	–
129	Flavone C-glycosides	C-hexosyl-luteolin *O*-feruloylpentoside	757.1	739	3.87	756.1	[M+H]^+^	–
130	Flavone C-glycosides	Chrysoeriol 8-*C*-hexoside	463.1	367.8	3.89	462.1	[M+H]^+^	–
131	Flavone C-glycosides	Chrysin *C-*hexoside	417.2	381.9	4	416.2	[M+H]^+^	–
132	Flavone C-glycosides	*C-p*entosyl-apigenin *O*-*p*-*coumaroylhexoside*	711.2	693.1	4.1	710.2	[M+H]^+^	–
133	Flavone C-glycosides	*C*-pentosyl-chrysoeriol 7-*O*-feruloylhexoside	771.2	177.5	4.25	770.2	[M+H]^+^	–
134	Flavone C-glycosides	8-*C*-hexosyl-apigenin *O-*feruloylhexoside	771.2	753	4.26	770.2	[M+H]^+^	–
135	Flavone C-glycosides	Apigenin 6*-C*-pentoside	403.1	367.7	4.3	402.1	[M+H]^+^	–
136	Flavone C-glycosides	Apigenin 8-*C*-pentoside	403.1	367.6	4.32	402.1	[M+H]^+^	–
137	Flavonol	Quercetin 5-*O*-malonylhexosyl-hexoside	713.1	713.1	2.96	712.1	[M+H]^+^	–
138	Flavonol	Dihydromyricetin	321.1	153.1	3.52	320.053	[M+H]^+^	C02906
139	Flavonol	Myricetin 3-*O*-galactoside	479.1	317.3	3.53	480.09	[M−H]^−^	–
140	Flavonol	Fustin	289	215.1	3.65	288.063	[M+H]^+^	C01378
141	Flavonol	Quercetin 7-*O*-rutinoside	611.2	303.7	3.7	610.2	[M+H]^+^	–
142	Flavonol	Kaempferol 3,7-dirhamnoside (Kaempferitrin)	579.2	433.1	3.74	578.164	[M+H]^+^	C16981
143	Flavonol	Kaempferol 3-*O*-robinobioside (Biorobin)	595.2	287	3.76	594.159	[M+H]^+^	–
144	Flavonol	Quercetin *O*-acetylhexoside	505.1	301.2	3.8	506.1	[M−H]^−^	–
145	Flavonol	Quercetin 4′-*O*-glucoside (Spiraeoside)	465	302.9	3.86	464.096	[M+H]^+^	–
146	Flavonol	Kaempferol 3-*O*-galactoside (Trifolin)	449	286.9	3.87	448.101	[M+H]^+^	C12626
147	Flavonol	Quercetin 3-*O*-glucoside (Isotrifoliin)	465	302.8	3.87	464.096	[M+H]^+^	C05623
148	Flavonol	Quercetin 3-*α*-*L*-arabinofuranoside (Avicularin)	435.1	303	3.98	434.0849	[M+H]^+^	–
149	Flavonol	Kaempferide	301.1	259.1	4.13	300.1	[M+H]^+^	C10098
150	Flavonol	Dihydroquercetin (Taxifolin)	303.1	125.1	4.15	304.058	[M−H]^−^	C01617
151	Flavonol	Isorhamnetin 5-*O-*hexoside	479.2	317.8	4.17	478.2	[M+H]^+^	–
152	Flavonol	Kaempferol 3-*O*-rhamnoside (Kaempferin)	431	285.1	4.48	432.106	[M−H]^−^	C16911
153	Flavonol	Aromadedrin (Dihydrokaempferol)	287.1	125.1	4.62	288.063	[M−H]^−^	C00974
154	Flavonol	Myricetin	319	319	4.7	318.038	[M+H]^+^	C10107
155	Flavonol	Quercetin-3,4′-*O-*di-β-glucopyranoside	627.1	465.2	4.83	626.1	[M+H]^+^	–
156	Flavonol	Kaempferol 7-*O*-rhamnoside	433.1	287	4.94	432.106	[M+H]^+^	–
157	Flavonol	Morin	301	151.1	5.11	302.04265	[M−H]^−^	C10105
158	Flavonol	Quercetin	303	303	5.13	302.043	[M+H]^+^	C00389
159	Flavonol	Laricitrin	333	305.2	5.15	332.053	[M+H]^+^	C12633
160	Flavonol	Kaempferol	285	214	5.73	286.048	[M−H]^−^	C05903
161	Flavonol	Syringetin	347	287.1	5.8	346.069	[M+H]^+^	C11620
162	Flavonol	Isorhamnetin	315.1	300.1	5.85	316.058	[M−H]^−^	C10084
163	Flavonol	Di-*O*-methylquercetin	329.1	314.3	5.91	330.1	[M−H]^−^	–
164	Flavonol	Ayanin	345.2	177.5	6.33	344.2	[M+H]^+^	C04444
165	Flavonol	Rhamnetin (7-*O*-methxyl quercetin)	317	317	6.43	316.058	[M+H]^+^	C10176
166	Flavonol	3,7-Di-*O*-methylquercetin	329	314	6.63	330.074	[M−H]^−^	C01265
167	Flavonol	Troxerutin (Trihydroxyethyl rutin)	347.3	285.4	6.66	346.251	[M+H]^+^	–
168	Flavonol	Kumatakenin	315.1	300	7.23	314.079	[M+H]^+^	–
169	Flavonolignan	Tricin 4′-*O-*(syringyl glyceryl)ether	557.2	331.9	4.44	556.2	[M+H]^+^	–
170	Flavonolignan	Tricin 7-*O*-*β*-guaiacylglycerol	527.1	331.7	5.44	526.1	[M+H]^+^	–
171	Flavonolignan	Tricin 4′*-O*-syringyl alcohol	497.1	331.8	5.75	496.1	[M+H]^+^	–
172	Flavonolignan	Tricin 4′-*O*-β-guaiacylglycerol	527.1	331.7	5.84	526.1	[M+H]^+^	–
173	Hydroxycinnamoyl derivatives	Caftaric acid	311.1	149.2	2.16	312.1	[M−H]^−^	–
174	Hydroxycinnamoyl derivatives	*O*-Caffeoyl maltotriose	665.1	323.4	2.23	666.1	[M+H]^+^	–
175	Hydroxycinnamoyl derivatives	Syringin	371.1	209.2	2.58	372.142	[M+H]^+^	C01533
176	Hydroxycinnamoyl derivatives	Coniferin	341	179.1	2.72	342.132	[M−H]^−^	C00761
177	Hydroxycinnamoyl derivatives	Caffeic acid *O*-glucoside	341	179.2	2.86	342	[M−H]^−^	–
178	Hydroxycinnamoyl derivatives	Homovanillic acid	181.1	137.1	3.04	182.0579	[M+H]^+^	C05582
179	Hydroxycinnamoyl derivatives	Hydroxy-methoxycinnamate	195.1	177.5	3.22	194.1	[M+H]^+^	–
180	Hydroxycinnamoyl derivatives	1-*O*-*β*-d-Glucopyranosyl sinapate	385.1	223.2	3.26	386.1	[M−H]^−^	–
181	Hydroxycinnamoyl derivatives	6-Hydroxymethylherniarin	207.1	147.4	3.31	206.1	[M+H]^+^	–
182	Hydroxycinnamoyl derivatives	Vanillic acid	169	111	3.35	168.042	[M+H]^+^	C06672
183	Hydroxycinnamoyl derivatives	Syringic acid	197.1	122.9	3.38	198.0528	[M−H]^−^	C10833
184	Hydroxycinnamoyl derivatives	Feruloyl syringic acid	375.2	137.6	3.47	374.2	[M−H]^−^	–
185	Hydroxycinnamoyl derivatives	*p*-Coumaryl alcohol	149	130.1	3.67	150.068	[M−H]^−^	C02646
186	Hydroxycinnamoyl derivatives	3-(4-Hydroxyphenyl)propionic acid	165.1	92.9	3.84	166.063	[M−H]^−^	C01744
187	Hydroxycinnamoyl derivatives	*p*-Coumaric acid	163	119	3.86	164.047	[M−H]^−^	C00811
188	Hydroxycinnamoyl derivatives	Coniferyl alcohol	179.1	146.1	3.88	180.079	[M−H]^−^	C00590
189	Hydroxycinnamoyl derivatives	Sinapyl alcohol	209	179.1	3.88	210.089	[M+H]^+^	C02325
190	Hydroxycinnamoyl derivatives	Ferulic acid	193.1	134.1	4.07	194.0579	[M+H]^+^	C01494
191	Hydroxycinnamoyl derivatives	3-Hydroxy-4-methoxycinnamic acid	193.1	134.1	4.08	194.0579	[M−H]^−^	–
192	Hydroxycinnamoyl derivatives	2-Methoxybenzoic acid	151	136.1	4.16	152.0473	[M−H]^−^	–
193	Hydroxycinnamoyl derivatives	*p*-Coumaraldehyde	149.1	131	4.42	148	[M−H]^−^	–
194	Hydroxycinnamoyl derivatives	Resveratrol	229.1	135	4.59	228.079	[M−H]^−^	C03582
195	Hydroxycinnamoyl derivatives	Sinapinaldehyde	207.1	177.1	4.61	208	[M−H]^−^	–
196	Hydroxycinnamoyl derivatives	Coniferylaldehyde	179.1	123	4.64	178.063	[M−H]^−^	C02666
197	Hydroxycinnamoyl derivatives	Pinoresinol	357.1	136.1	5.41	358.142	[M+H]^+^	–
198	Hydroxycinnamoyl derivatives	4-Methoxycinnamic acid	177	145.2	5.5	178	[M−H]^−^	–
199	Hydroxycinnamoyl derivatives	3,4-Dimethoxycinnamic acid	207.1	192.1	5.52	208.1	[M−H]^−^	–
200	Hydroxycinnamoyl derivatives	*trans*-cinnamaldehyde	133.1	115	5.97	132.0575	[M+H]^+^	C00903
201	Hydroxycinnamoyl derivatives	Caffeic aldehyde	165.1	95.5	6.04	164.1	[M+H]^+^	C10945
202	Hydroxycinnamoyl derivatives	Syringaldehyde	183.1	165.5	6.58	182.1	[M−H]^−^	–
203	Hydroxycinnamoyl derivatives	Methyleugenol	179	138	7.3	178.099	[M−H]^−^	C10454
204	Hydroxycinnamoyl derivatives	Caffeyl alcohol	317.2	281.3	7.47	316	[M+H]^+^	C09066
205	Isoflavone	Daidzein 7-*O*-glucoside (Daidzin)	417.1	255.1	3.36	416.111	[M+H]^+^	C10216
206	Isoflavone	Glycitin	445	282.1	3.54	446.121	[M+H]^+^	C16195
207	Isoflavone	Genistein 7-*O*-Glucoside (Genistin)	433	270.9	4.01	432.106	[M−H]^−^	C09126
208	Isoflavone	Formononetin 7-*O*-glucoside (Ononin)	429.1	267.1	4.59	430.126	[M+H]^+^	C10509
209	Isoflavone	2′-Hydroxygenistein	287	217.1	4.89	286.048	[M−H]^−^	C12134
210	Isoflavone	Daidzein	255.1	199.1	4.89	254.0579	[M+H]^+^	C10208
211	Isoflavone	Orobol (5,7,3′,4′-tetrahydroxyisoflavone)	285	257.1	5.08	286.048	[M−H]^−^	C10510
212	Isoflavone	Sissotrin	447.1	285.1	5.16	446.121	[M−H]^−^	C05376
213	Isoflavone	Formononetin (4′-*O*-methyldaidzein)	269.1	269.1	6.33	268.074	[M+H]^+^	C00858
214	Isoflavone	Prunetin	283	268.1	6.97	284.069	[M−H]^−^	C10521
215	Phenolamides	Spermidine	146.2	72.1	0.56	145.2	[M+H]^+^	C00315
216	Phenolamides	Spermine	203	112	0.62	202	[M+H]^+^	C00750
217	Phenolamides	Putrescine	89	71.9	0.64	88.1	[M+H]^+^	C00134
218	Phenolamides	Agmatine	131.1	72.1	0.75	130.1	[M+H]^+^	C00179
219	Phenolamides	1,5-Diaminopentane	103	86.1	0.76	102.116	[M+H]^+^	C01672
220	Phenolamides	*N*-Acetylputrescine	131	71.9	0.8	130.11061	[M+H]^+^	C02714
221	Phenolamides	*N*-hexosyl-*p*-coumaroyl putrescine	397.1	147.4	1.73	396.1	[M+H]^+^	–
222	Phenolamides	*N*-Caffeoyl putrescine	251.1	233.5	1.97	250.1	[M+H]^+^	C03002
223	Phenolamides	*N*-(4′-*O*-glycosyl)-*p*-coumaroyl agmatine	439.1	147.4	2.11	438.1	[M+H]^+^	–
224	Phenolamides	*N*′,*N*″,*N*″′-*p*-coumaroyl-cinnamoyl-caffeoyl spermidine	584.2	325.8	2.42	583.2	[M+H]^+^	–
225	Phenolamides	*N*′, *N*″-di-p-coumaroylspermine	495.3	478.4	2.43	494.3	[M+H]^+^	–
226	Phenolamides	*N*′-Feruloyl putrescine	265.1	177.5	2.44	264.1	[M+H]^+^	–
227	Phenolamides	*N*-Caffeoyl agmatine	293.2	234.5	2.45	292.2	[M+H]^+^	–
228	Phenolamides	*N*-Sinapoyl putrescine	295	207.6	2.77	294	[M+H]^+^	–
229	Phenolamides	*N*′, *N*″-disinapoylspermidine	558.3	264.8	3.75	557.3	[M+H]^+^	–
230	Proanthocyanidins	Procyanidin B3	577.1	407.3	2.79	578.1424	[M−H]^−^	–
231	Proanthocyanidins	Procyanidin A3	577.1	425.4	2.92	576.1	[M+H]^+^	–
232	Proanthocyanidins	Procyanidin B2	579.1	127.3	3.03	578.1424	[M+H]^+^	–
233	Proanthocyanidins	Procyanidin A1	575	285.3	3.68	576.1268	[M−H]^−^	–
234	Proanthocyanidins	Procyanidin A2	577	425.9	4.06	576.1268	[M+H]^+^	C10237
235	Quinate and its derivatives	Quinic acid	191	85	0.92	192.063	[M−H]^−^	C00296
236	Quinate and its derivatives	*p*-Coumaroyl quinic acid *O-*glucuronic acid	513.1	191.2	1.82	514.1	[M−H]^−^	–
237	Quinate and its derivatives	Quinacyl syringic acid	371.1	179.2	1.85	372.1	[M−H]^−^	–
238	Quinate and its derivatives	Homovanilloyl quinic acid	355.1	181.2	1.89	356.1	[M−H]^−^	–
239	Quinate and its derivatives	5-*O*-*p*-coumaroyl quinic acid *O*-hexoside	499.1	163.2	2.1	500.1	[M−H]^−^	–
240	Quinate and its derivatives	*O*-Feruloyl quinic acid	369.1	177.5	2.12	368.1	[M+H]^+^	–
241	Quinate and its derivatives	Quinic acid *O*-di-glucuronic acid	543.1	191.2	2.13	544.1	[M−H]^−^	–
242	Quinate and its derivatives	Neochlorogenic acid (5-*O*-Caffeoylquinic acid)	353	191.1	2.35	354.095	[M−H]^−^	C17147
243	Quinate and its derivatives	1-*O*-Caffeoyl quinic acid	353.1	191.1	2.38	354.095	[M−H]^−^	–
244	Quinate and its derivatives	3-*O*-*p*-coumaroyl quinic acid *O*-hexoside	499.2	173.2	2.45	500.2	[M−H]^−^	–
245	Quinate and its derivatives	5-*O*-*p*-coumaroyl shikimic acid *O*-hexoside	481.1	445.4	2.62	482.1	[M−H]^−^	–
246	Quinate and its derivatives	Chlorogenic acid (3-*O*-Caffeoylquinic acid)	353.1	191.1	2.72	354.0951	[M−H]^−^	C00852
247	Quinate and its derivatives	1-*O*-*p*-Coumaroyl quinic acid	337.1	155.8	2.85	338.1	[M−H]^−^	–
248	Quinate and its derivatives	4-*O*-Caffeoyl quinic acid (criptochlorogenic acid)	353.1	191.2	3	354.1	[M−H]^−^	–
249	Quinate and its derivatives	3-*O*-Feruloyl quinic acid	369.1	177.5	3.01	368.1	[M+H]^+^	C02572
250	Quinate and its derivatives	*O*-Sinapoyl quinic acid	399	207.5	3.17	398	[M+H]^+^	–
251	Quinate and its derivatives	3-*O*-*p*-coumaroyl shikimic acid *O*-hexoside	481.1	319.3	3.23	482.1	[M−H]^−^	–
252	Quinate and its derivatives	5-*O*-*p*-Coumaroylquinic acid	337	275.8	3.26	338	[M−H]^−^	–
253	Quinate and its derivatives	3-*O*-*p*-Coumaroyl quinic acid	337.1	190.9	3.3	338.1	[M−H]^−^	–
254	Quinate and its derivatives	1-*O*-Feruloyl quinic acid	369.1	207.5	3.42	368.1	[M+H]^+^	–
255	Quinate and its derivatives	Chlorogenic acid methyl ester	367	179.1	3.64	368.111	[M−H]^−^	–
256	Quinate and its derivatives	5-*O*-*p*-Coumaroyl shikimic acid	321.1	147.5	3.81	320.1	[M+H]^+^	–
257	Quinate and its derivatives	3-*O*-*p*-Coumaroyl shikimic acid	319	145.3	3.93	320	[M−H]^−^	–

### Absolute quantification of catechin derivatives and total proanthocyanidins (PAs) contents

**Figure 3:**
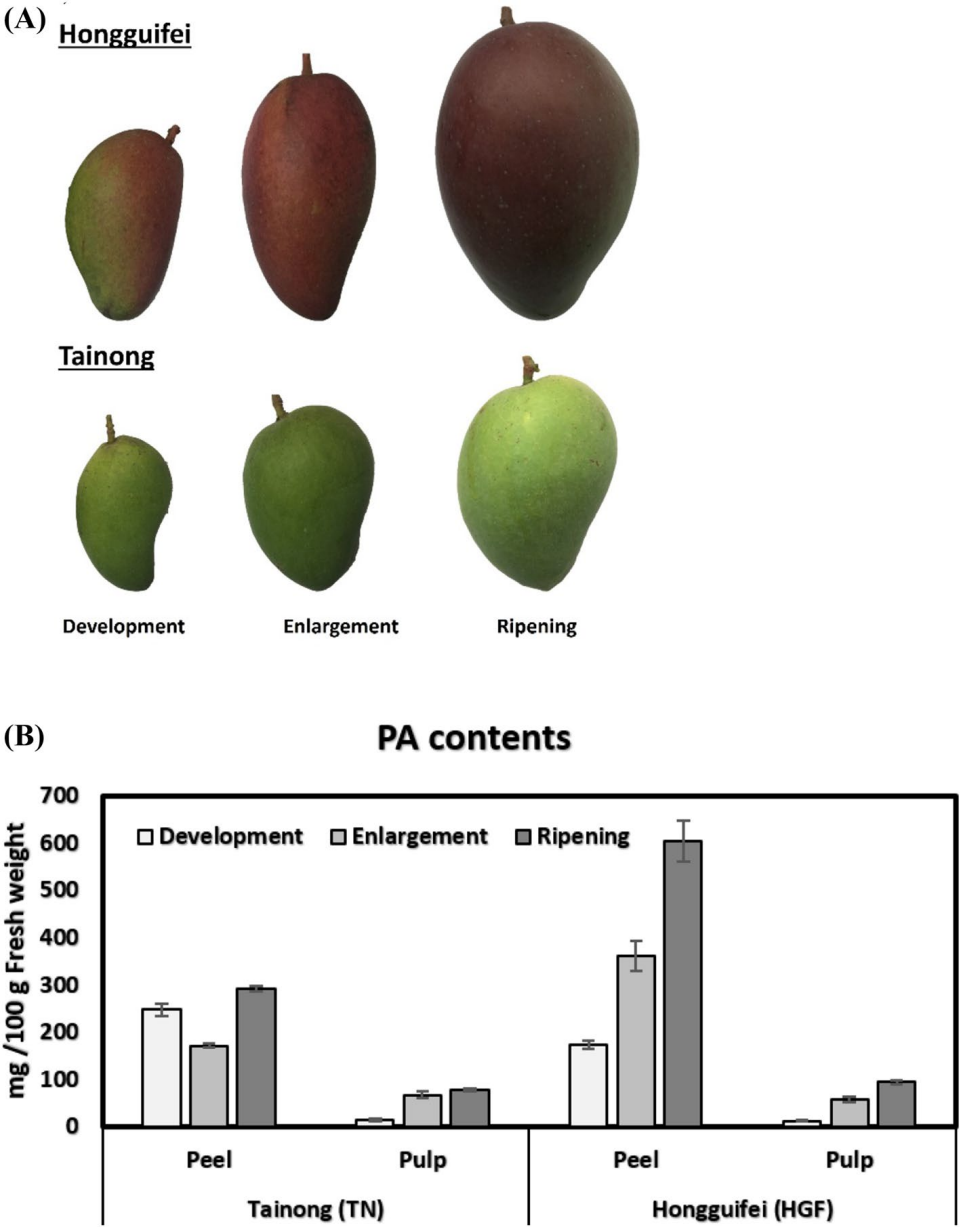
Fruit color and total proanthocyanidin (PA) contents of Hongguifei (HGF) and Tainong (TN) mango.

Current study reports developmental stage dependent variation (Fig. 3A) in total proanthocyanidins (PAs) contents using a method described by Dong et al.^34^. Pulp samples of both cultivars contain similar quantities of PAs. However, there is an increasing trend for PAs contents towards fruit maturity in both cultivars (Fig. 3B). Fruit peels of both cultivars contain higher amount of PAs as compared to pulps. For TN cultivar, the concentration of peel PAs remained in close range across three growth stages. However, there was a massive increase in PAs content of HGF cultivar towards maturity (Fig. 3B). Very few studies have documented the concentration of PAs in mango and it is often influenced by several factors including tissue type, geographical area and method of extraction^33,35–37^. Pulp of mangoes from USA were reported to contain 12.8 mg proanthocyanidins/100 g fresh weight. Two different extraction methods yielded 0.18 and 0.48 mg PAs per 100 g DW of mangoes from Spain^36^. Similarly, procyanidins A2 (14–78 µg /ml), B1 (29–88 µg /ml) and B2 (0–10 µg /ml) were reported in peel liqueurs of mangoes from Brazil^37^.

The phenolic compounds are among the major contributors that are accountable for antioxidant properties in fruits, vegetables, whole grains and other plant-based materials^38^. The TN mango fruit contains higher levels of total phenolics (TPs) and total flavonoids as compared to HGF^2^. However, total anthocyanin contents (TAs) were reported to be higher in HGF as compared to TN^2^. In phenylpropanoid biosynthesis pathway, both anthocyanins and proanthocyanins belong to the terminal steps^39^. Therefore, the increase in PAs (Fig. 3) potentially correlates with anthocyanins^2^ in HGF mango.

**Figure 4:**
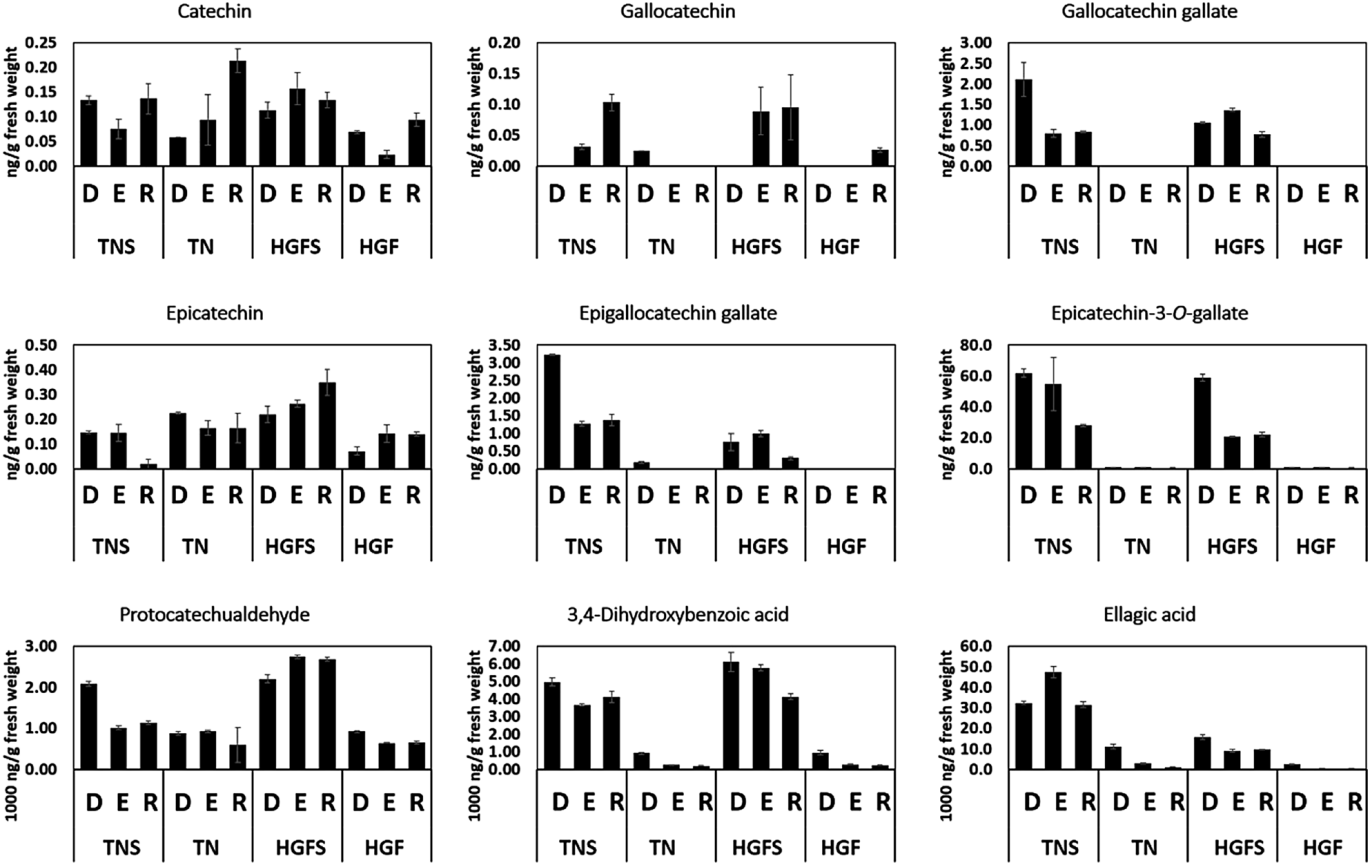
HPLC based quantification of Catechin, derivatives and phenolic acids in the pulp and peel of both cultivars. TNS and HGFS represent quantification in peel of respective cultivars. TN and HGF represent quantification in pulp of respective cultivars. The letters D, E and R represent development, enlargement and ripening of mango fruit. The use of commercial standards for the quantification of catechin derivatives and phenolic acids along with linear equations, correlation coefficients, LOD’s, and LOQ’s values are presented in Table S5.

In order to validate the results of metabolome based estimation of relative quantities of catechin and derivatives, absolute quantities of catechin and its derivatives were calculated in peel and pulp samples of both cultivars using HPLC. It was observed that the concentrations of catechin, gallocatechin, gallocatechin gallate, epicatechin, epicatechin gallate, epicatechin-3-*O*-gallate, protocatechuic aldehyde, 3,4-Dihydroxybenzoic acid and ellagic acid were differentially present in both cultivars for pulp and peel samples (Fig. 4). Overall, peel samples contained higher amounts of these metabolites that decreased with the age of fruit^40^. Moreover, fold change modifications of these metabolites follow similar trend (with minor variations) as discussed above (Table S2). In previous studies, these compounds were either individually reported as catechine^41^, epicatechin^42^, protocatechuic acid^2^ and ellagic acid or in terms of total proanthocyanidin and tannins^10^. The composition of phenolic compounds in peels has attracted a crucial importance for mango in calculating functional food mixtures^43^.

### Differential expression of *MiANR* and *MiLAR* genes

**Figure 5:**
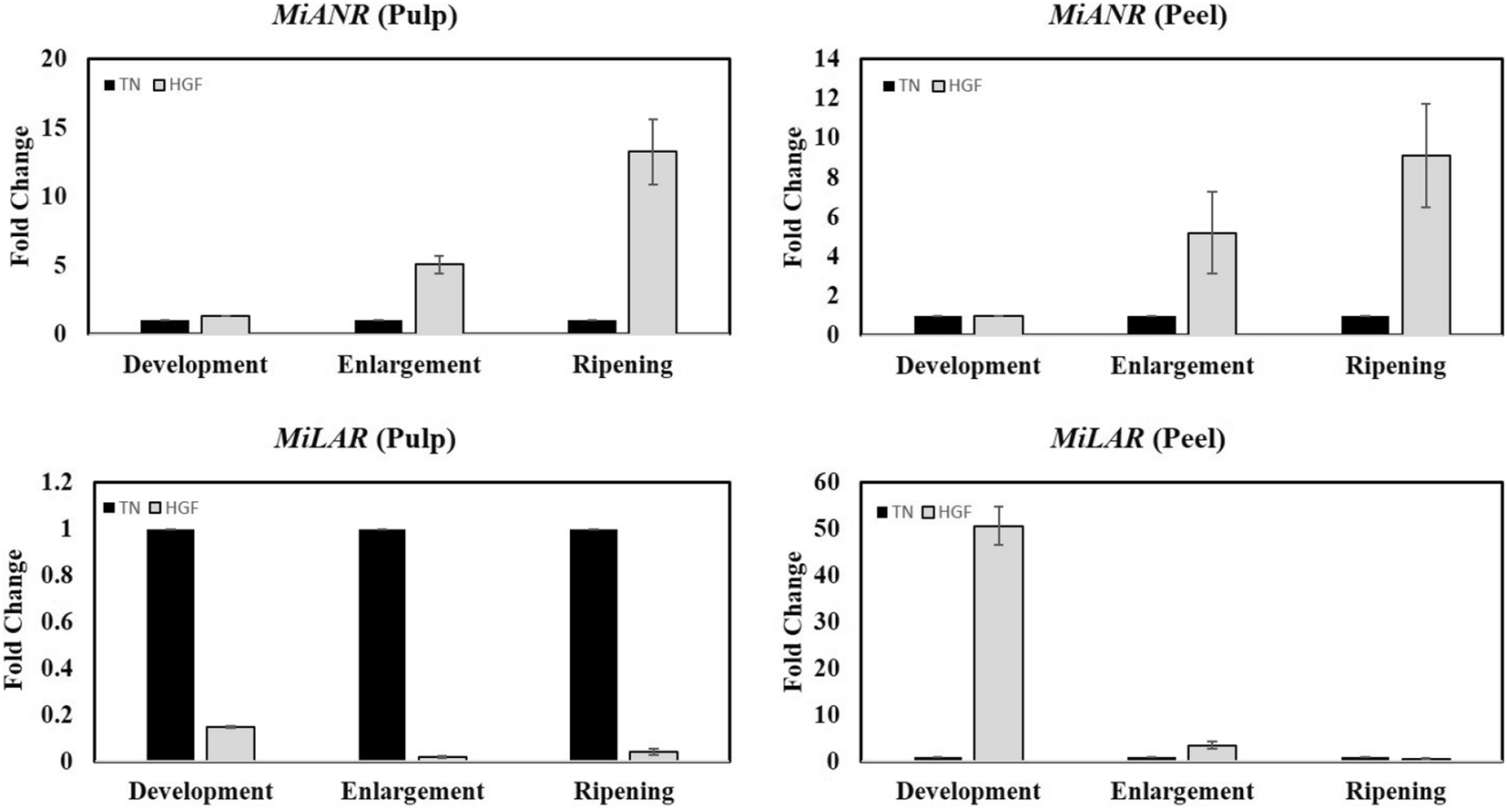
Relative RT-qPCR analysis of *MiANR* and *MiLAR* gene.

The genes encoding anthocyanidin reductase (ANR) and leucoanthocyanidin reductase (LAR) enzyme have been cloned and characterized in plants including poplar, buckwheat, lotus and fruits such as grapevine, strawberry, persimmon, apple, and mango (for references see^44^). The expression patterns of these genes are highly correlated with PA accumulation in many plants. To determine whether the differential expression of catechin/derivatives correlated with the transcript abundance of *MiANR* and *MiLAR,* the expression levels of these genes were analyzed in fruits of both cultivars using relative qRT-PCR (Fig. 5). In pulp samples, both genes exhibited an opposite expression profile i.e., the expression of *MiANR* increased with fruit maturity in HGF and *MiLAR* followed similar pattern in TN cultivar. In peel samples of both cultivars, the relative difference of expression increased (with fruit maturity) for *MiANR* and decreased (with fruit maturity) for *MiLAR* (Fig. 5). These results suggested that transcription of *LAR* and *ANR* seems controlled by a feedback mechanism^45^. It means a higher concentration of catechin may stimulate the expression of *LAR* and higher levels of epicatechin can promote the expression of *anthocyanidin synthase* (*ANS*) and *ANR*. In addition, there could be a potential competition between LAR and ANR enzymes. The activity of both of these reductases (ANR and LAR) is dependent on NAPDH. Therefore, if one of them is overexpressed, it will decrease the availability of NAPDH for the other enzyme. It is expected that such competition may govern the mutual inhibition of *LAR* and *ANR* expression observed in this study. Altogether, the PA biosynthesis is likely co-regulated by structural genes such as LAR and ANR, and the mutual inhibition between the ANR and LAR expression may affect PA accumulation^16^. A lack of clear association between the transcripts of *LAR*/*ANR* and the catechin/derivatives has already been reported in other plants as well^40,46^. Earlier studies indicate a potential importance of *ANR* and *LAR* genes in the biosynthesis of galloylated catechins^47^. Therefore, it could be explained in terms of variable amounts of catechin/derivatives and the fact that different forms of catechin are potentially interconvertible.

In each comparison, the default expression value of both genes was adjusted to one in samples obtained from TN cultivar.

## Materials and methods

### Plant material, sample preparation and extraction

Current study compares metabolites of fruit peel and pulp of two mango varieties named Tainong (TN) and Hongguifei (HGF), during three different developmental stages of fruit (Development or 1st, Enlargement or 2nd, and Ripening or 3rd). Both varieties were maintained at a farm located at Basuo town, Dongfang County, Hainan province. The TN mango was developed by the Fengshan tropical horticulture branch institute of the Taiwan Agricultural Test Institute and introduced to southern provinces of China including Hainan and Guangdong in 1994. The TN fruit is oval-shaped and relatively small in size that weighs up to 300 g. Mature fruit peel is dark green to greenish-yellow. The peel is slightly thicker and ductile, which is good for storage and transportation The HGF mango, also known as Hongjinglong, is native to Taiwan. It is a hybrid of 'Irwin' and 'Kate' and was introduced to Hainan in the 1990s^48,49^. The HGF mango fruit is oblong, the top of the fruit is small and the fruit is relatively large (300–500 g). Mature fruit peel is purple-red and green (Fig. 3A).

Fruit samples from both varieties were collected at 40 (Developmental or 1st), 65 (Enlargement or 2nd), and 90 (Ripening or 3rd) days after full bloom (DAFB) respectively. Samples for each stage consisted of 10 fruits from 10 mango trees. Fruits of each variety were peeled and cored, and the flesh was cut into small sections. Fruit samples at each stage were mixed and immediately frozen in liquid nitrogen, and then stored at − 80 °C until used.

A mixer mill with zirconia bead (MM 400, RETSCH) was used to grind the freeze-dried samples for 90 s at 30 Hz. Then, an overnight extraction (at 4 °C using 70% aqueous methanol) was performed for 100 mg powder. Before LC–MS analysis, the samples were centrifuged at 10,000×*g* for 10 min followed by filtration (SCAA-104, 0.22 μm pore size; ANPEL, Shanghai, China, https://www.anpel.com.cn/).

### HPLC analysis

An LC–ESI–MS/MS system (HPLC, Shim-pack UFLC SHIMADZU CBM30A system, www.shimadzu.com.cn/; MS, Applied Biosystems4500 Q TRAP, www.appliedbiosystems.com.cn/) was used to analyze the sample extracts. The analytical parameters were as follow: HPLC column, waters ACQUITY UPLC HSS T3 C18 (1.8 µm, 2.1 mm * 100 mm); solvent system, water (0.04% acetic acid): acetonitrile (0.04% acetic acid); gradient program, 100:0 V/V at 0 min, 5:95 V/V at 11.0 min, 5:95 V/V at 12.0 min, 95:5 V/V at 12.1 min, 95:5 V/V at 15.0 min; flow rate, 0.40 mL/min; temperature, 40 °C; injection volume: 5 μl. The effluent was alternatively connected to an ESI-triple quadrupole-linear ion trap (Q TRAP)-MS.

### ESI-Q TRAP-MS/MS

Triple quadrupole-linear ion trap mass spectrometer (QTRAP; API 4500 Q TRAP LC/MS/MS System) was used for Linear Ion Trap (LIT) and triple quadrupole (QQQ) scans. The equipment contained an ESI Turbo Ion-Spray interface, which was operated in a positive and negative ion-mode and the data was analyzed using analyst 1.6 software (AB SCIEX). The chromatographic method (e.g., mobile phase composition, pH, elution gradient) is the same in both ESI acquisition modes. Following conditions were used for the source of ESI operation: turbo spray (ion-source); 550 °C (source temperature); 5500 V (ion spray voltage or IS); GSI (ion source gas I), GSII (gas II), CUR (curtain gas) were set at 55, 60, and 25.0 psi, respectively; CAD (the collision gas) was set at high. Polypropylene glycol (10 and 100 μmol/L) was used to tune the instrument and for calibration of mass in QQQ and LIT modes, respectively. The collision gas (N_2_) was set to 5 psi during QQQ scans based MRM analysis. A specific set of MRM transitions were monitored for each period according to the metabolites eluted within this period.

### Quantitative and qualitative principles of metabolites

Based on the public database and the self-built database MWDB (metware) of metabolite information, the first-order spectrum and two-level spectral data of spectral detection were qualitatively analyzed. The structural analysis of metabolites is referenced by knapsack (https://kanaya.naist.jp/KNApSAcK/), Massbank (https://www.massbank.jp/), Metlin (https://metlin.scripps.edu/index.php), MoTo DB (https://www.ab.wur.nl/moto/), hmdb (https://www.hmdb.ca/), and other existing mass spectrometry public databases. The quantification of metabolites was accomplished using the multi-reaction monitoring model of the triple four-stage rod mass spectrometry (multiple reaction monitoring, MRM). The detection standard of MRM is based on the parameters including Q1, Q3, RT, DP, CE from the database, which was built using the standards. The relative content of the compounds was determined by the signal intensity of Characteristic fragment ion Q3. The range of DP (declustering potential) was − 80 to 80 V and CE (collision energy) was − 50 to 50 V. In MRM mode, the four levers first filter the precursor ions of the target, the ions matching substances with different molecular weights are excluded for initial removal of disturbance, and the precursor ions are induced by the collision chamber to form many fragments of ions. The fragment ions are then filtered through the triple four-pole filter to select characteristic fragment ions, eliminate non-target ion interference, make the quantification more accurate, and improve repeatability. After obtaining the data of the different samples of the metabolite spectra, the peak area integral of all the material mass spectra was obtained, and the mass spectra of the same metabolites in different samples were corrected by integral correction.

### Statistical analysis

The SIMCA14.1 software package (V14.1, Sartorius Stedim Data Analytics AB, Umea, Sweden) was used for principal component analysis (PCA) and orthogonal projections to latent structures-discriminate analysis (OPLS-DA). PCA showed the distribution of the original data. In order to obtain a higher level of group separation and to get a better understanding of variables responsible for classification, supervised OPLS-DA were applied. Based on OPLS-DA, a loading plot was constructed, which showed the contribution of variables to differentiate between two groups. The first principal component of variable importance in the projection (VIP) was calculated to refine this analysis. The VIP values above one were designated as differential metabolites. In the second step, Student’s t-test was used to assess the remaining variables and variables with *p* value > 0.05 were discarded between two comparison groups. In addition, commercial databases including KEGG^50^ https://www.genome.jp/kegg/ and MetaboAnalyst https://www.metaboanalyst.ca/ were used to search for the pathways of metabolites.

### Determination of total proanthocyanidins (PAs) content

The proanthocaynidins content of the extracts were determined using the method described by DongRuixia[12]. Calibration curve was prepared by mixing methanol solution of standard proanthocyanidin (1 mL; 0.2–1 mg/mL) with 6 mL of 4% (g/v) vanillic aldehyde and 3 mL of concentrated HCl. After capping and shaking the tube, it was incubated in the dark for 15 m at 30℃ ± 1. The absorbance was measured at 500 nm (UVmini-1240, Shimadzu Corporation, Kyoto, Japan) with methanol as blank control and the standard curve was plotted. 1 mL of each of the extract solution in methanol (0.1 g mL^−1^) was also mixed with the above mentioned reagents, After incubation for 30 min, the absorbance was measured to determine proanthocyanidins content. The concentration of proanthocyanidin of samples was calculated using the following equation based on a PAs standard curve: y = 1.038x + 0.046 Where X is the absorbance and y is the proanthocyanidin equivalent. For the precentage of PAs content in tested samples, the equation is as follow: D = (v Cn/1000 W) 100%. All tests were conducted in triplicate D: the percentage of PAs content of samples; V: The constant volumn of sample; C: the concentration of proanthocyanidin of samples (mg/mL); n: dilution times; W: weight of sample(dry weight). Then the percentage of PAs conent of samples was converted to mg/ 100 g FW based on the ratio of fresh and dry weight of mango.

### RNA extraction and relative RT-qPCR analysis

Peel and pulp samples were used for RNA extraction with the help of RNAprep Pur Plant Kit for polysaccharides and polyphenolics-rich samples (TIANGEN Biotech, Beijing) following the instructions of the manufacturer. The concentration of RNA was estimated from each sample through NanoDrop spectrophotometer (BERTHOLD, Germany). All-in-one First-Strand Synthesis Mastermix, with DNaseI (NOVA BIOMED, China) was used to reverse transcribe 1.0 μg of total RNA. The qRT-PCR analysis was performed using an APPLIED BIOSYSTEMS StepOnePlus Real-Time PCR System and TB GREEN Premix Ex Taq II, Tli RNaseH plus kit (TAKARA). The Oligo Calculator (https://mcb.berkeley.edu/labs/krantz/tools/oligocalc.html) was used for designing the gene-specific primers and NCBI Primer-BLAST program (https://www.ncbi.nlm.nih.gov/tools/primer-blast/) was used to verify primer specificity. The expression data were normalized using *MiActin* gene (GenBank accession number HQ830244) as an internal control. Following primers were used for RT-qPCR: *MiANR* (Fow-TCCAAGACCCTGGCTGAAAG; Rev-CTGGCGTAAGAGAAGGACCA), *MiLAR* (Fow-ATTAAACCAGCTCCCTCTCG; Rev-CACATCATGCCCAAACTCAG), and *MiActin* (Fow-GCTTGCCTATGTTGCCCTTGACTA; Rev-GCATCGGAATCTCTCAGCTCCAAT). An equal amount of cDNA template was used for each sample including the internal control. The qPCR analysis was repeated in three independent experiments.

### Ethical approval

This article does not contain any studies with human participants or animals performed by any of the authors.

## Conclusions

The current study provides a global picture of metabolite dynamics between two mango cultivars by conducting an analysis of widely-targeted metabolomics based on LC–MS/MS data. The differential accumulation or absence of particular metabolites from either of cultivars indicates underlying differential metabolism. Important metabolites including catechin, its derivatives and procyanidins only up-regulated in HGF pulp and peel samples. The current study revealed that the expression of *MiANR* (a key gene of the PP pathway) was significantly higher in both pulp and peel samples of HGF cultivar at all three stages of fruit development. Moreover, total proanthocyanidin contents and relative flavan-3-ols/procyanidins were also higher in HGF cultivar. It seems obvious that such variations are directly responsible for the detected differences in relative quantities of flavonoid. This study documented changes in absolute contents of important catechin/ derivatives and expression profile of key genes involved in their biosynthesis for three growth stages. Such knowledge of mango fruit will be helpful for producers in adding value to the fruit and increasing antioxidant components. Moreover, different derivatives show distinct contents towards maturity and higher concentrations in peels as compared to pulp. It advocates the need for further research to improve contents of these metabolites in edible portion of mango.

## Supplementary information

Supplementary Figure 1.

Supplementary Figure 2A.

Supplementary Figure 2B.

Supplementary Table 1.

Supplementary Table 2.

Supplementary Table 3.

Supplementary Table 4.

Supplementary Table 5.

Supplementary Legends.
