# Supplementary figures and images for: Comprehensive ESI-Q TRAP-MS/MS based characterization of metabolome of two mango (Mangifera indica L) cultivars from China
Source: Sci Rep. 2020 Nov 18;10:20017. doi: 10.1038/s41598-020-75636-y (PMC7676270; doi:10.1038/s41598-020-75636-y)

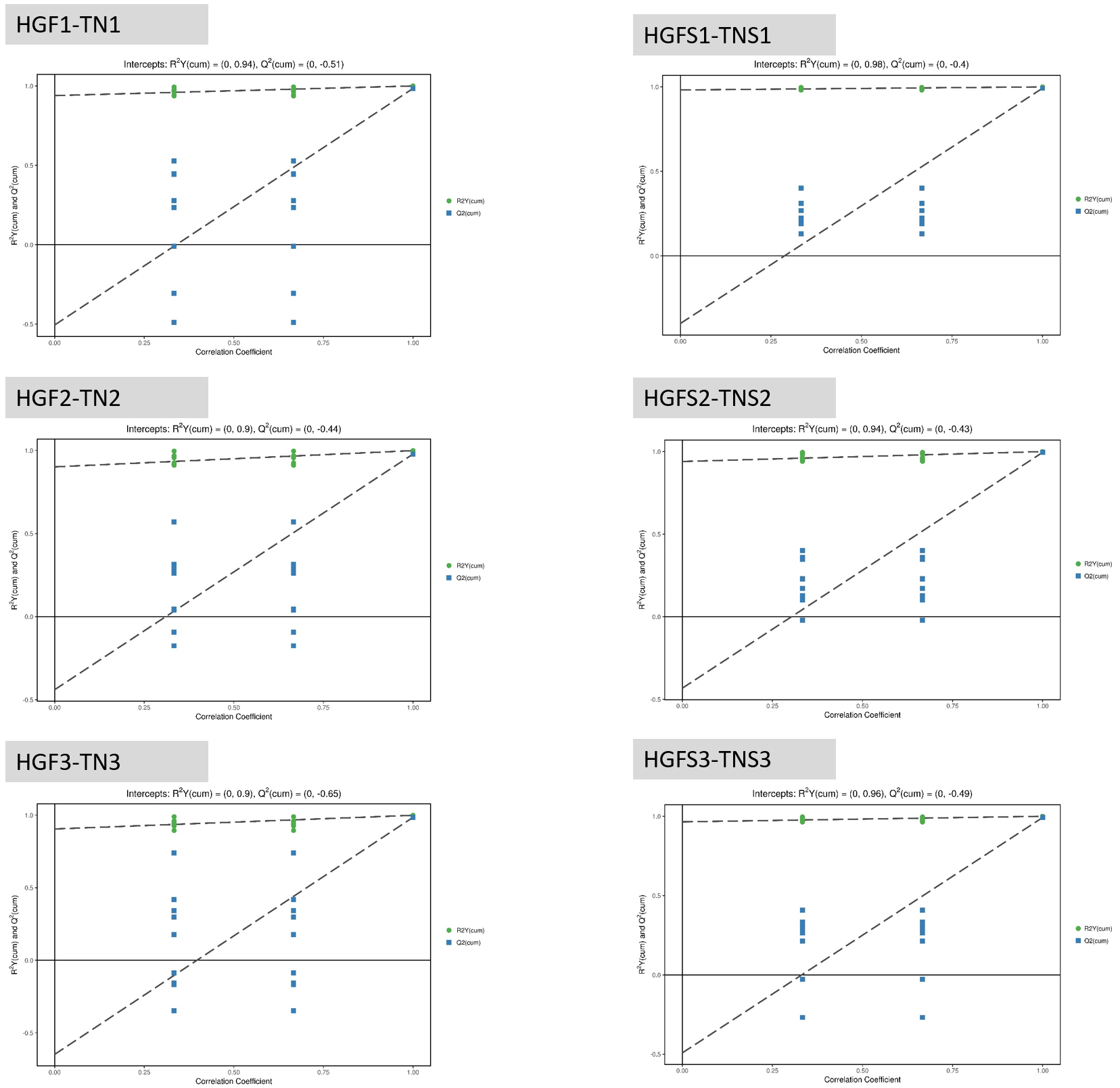

Supplement: Supplementary file 1 — Supplementary Figure 1. [file 41598_2020_75636_MOESM1_ESM.tif]

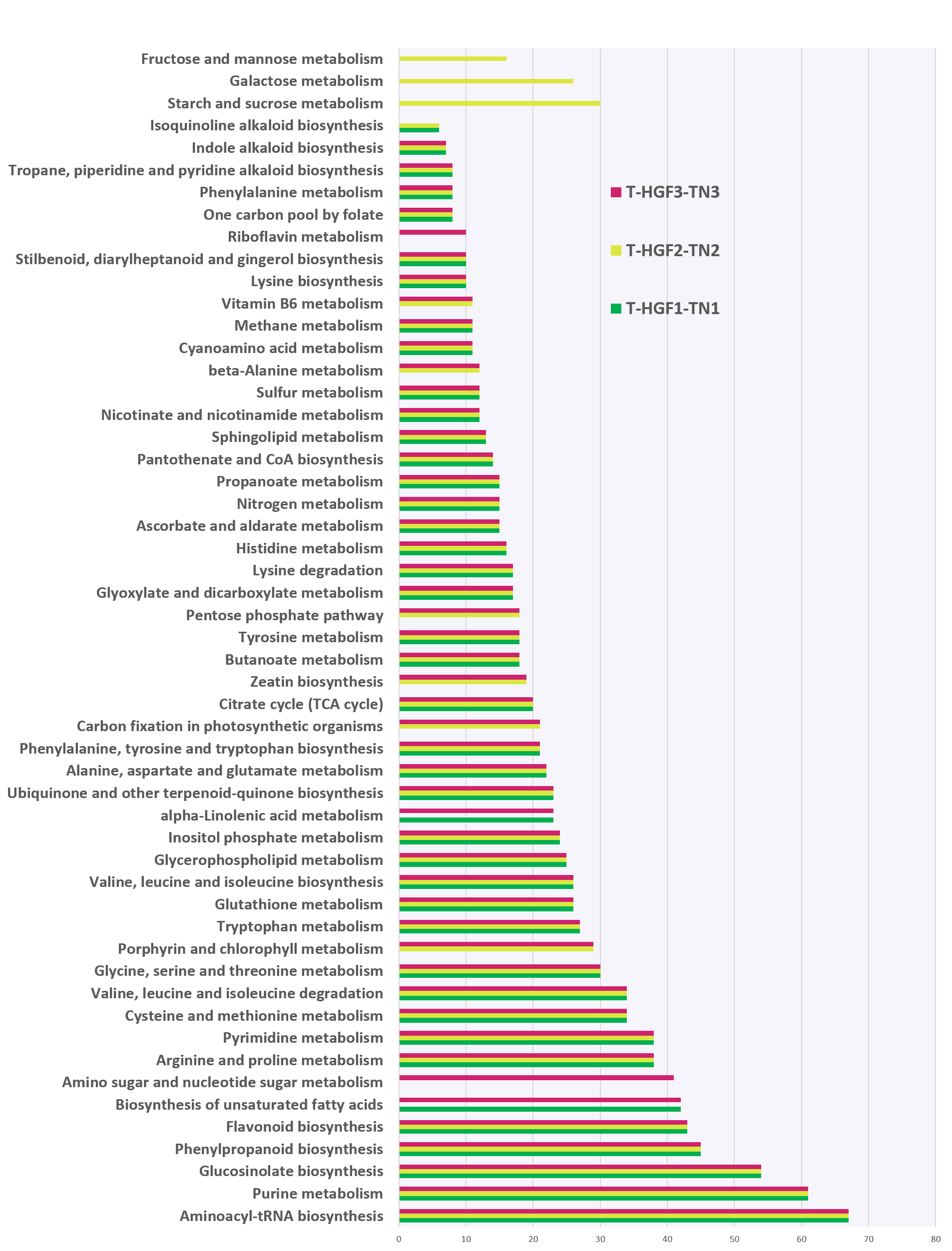

Supplement: Supplementary file 2 — Supplementary Figure 2A. [file 41598_2020_75636_MOESM2_ESM.tif]

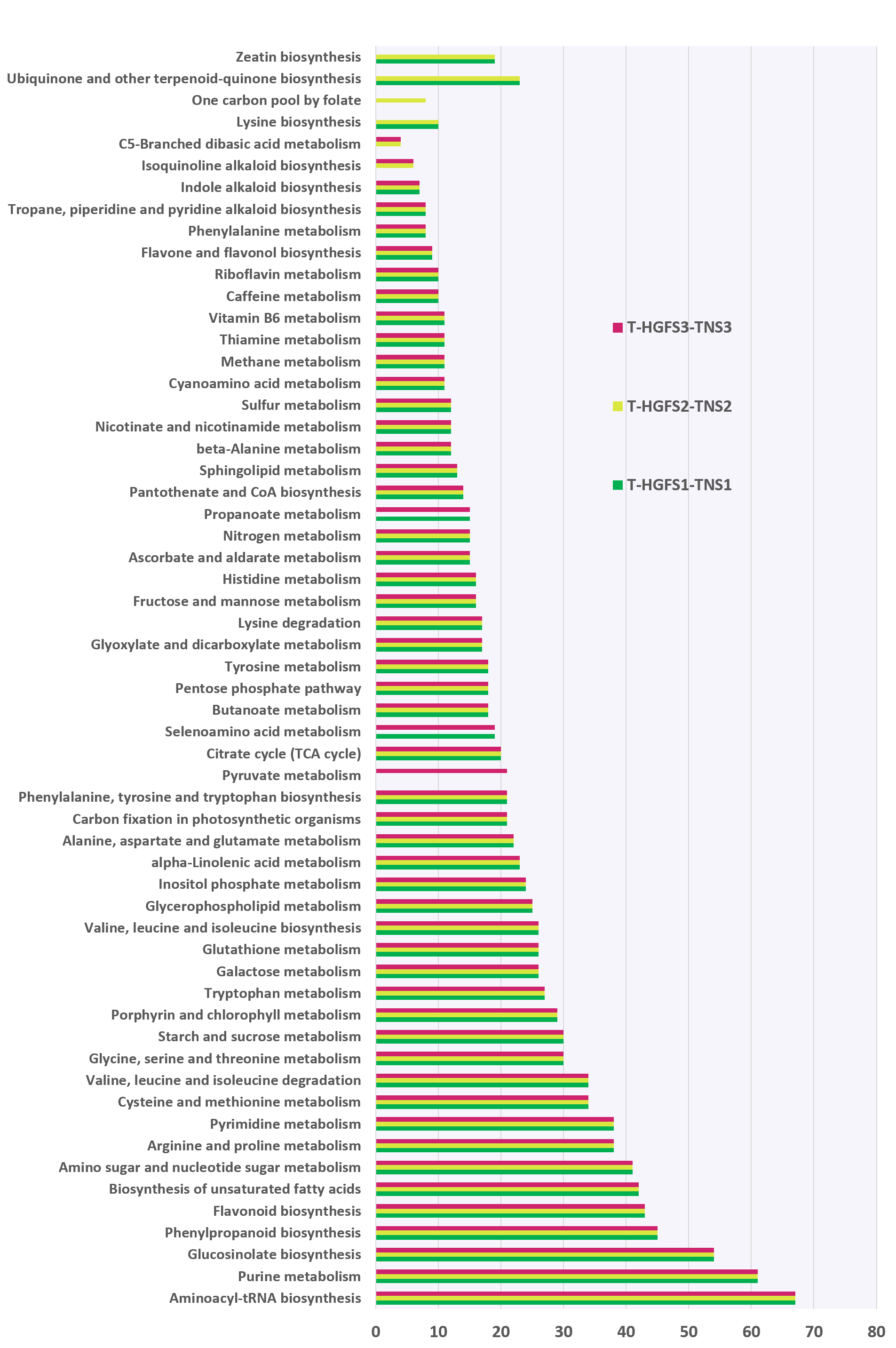

Supplement: Supplementary file 3 — Supplementary Figure 2B. [file 41598_2020_75636_MOESM3_ESM.tif]
