# Supplementary material for: Comprehensive ESI-Q TRAP-MS/MS based characterization of metabolome of two mango (Mangifera indica L) cultivars from China
Source: Sci Rep. 2020 Nov 18;10:20017. doi: 10.1038/s41598-020-75636-y (PMC7676270; doi:10.1038/s41598-020-75636-y)
Supplement: Supplementary file 9 — Supplementary Legends. [file 41598_2020_75636_MOESM9_ESM.docx]

**Supplementary Materials:**

**Supplementary Fig. S1:** OPLS-DA permutation plots

**Supplementary Fig. S2:** Metabolic Pathways

**Supplementary Table S1:** List of metabolites detected in the current study

**Supplementary Table S2:** Differentially expressed metabolites

**Supplementary Table S3:** PCA and OPLS-DA models

**Supplementary Table S4:** Differential metabolic pathways between both cultivars

**Supplementary Table S5:** The use of commercial standards for the quantification of catechin derivatives and phenolic acids
